# Supplementary material for: Decay and renormalization of a longitudinal mode in a quasi-two-dimensional antiferromagnet
Source: Nat Commun. 2021 Sep 9;12:5331. doi: 10.1038/s41467-021-25591-7 (PMC8429660; doi:10.1038/s41467-021-25591-7)
Supplement: Supplementary file 1 — Supplementary information [file 41467_2021_25591_MOESM1_ESM.pdf]

# Supplementary Information

## Supplementary Note 1. Single-ion state of $Fe^{2+}$ in $Ba_2FeSi_2O_7$

Recent terahertz spectroscopy and X-ray absorption spectroscopy (XAS) on $A_2FeSi_2O_7$ (A=Sr and Ba) revealed that the considerable tetragonal distortion of $FeO_4$-tetrahedra (Sr: 17% and Ba: 26% $z$-compression from cubic) with large spin-orbit coupling of $Fe^{2+}$ ($\lambda \sim$20 meV) induces significant easy-plane single-ion anisotropy in the system[1,2]. Following the description for the spin-orbital states of $Fe^{2+}$ in a tetrahedral environment, in $Ba_2FeSi_2O_7$ the large single-ion anisotropy determines the ground state from the multiplet of $Fe^{2+}$ ion. Starting with the free ion, $^5D$ ($L = 2, S = 2$), the tetrahedral crystal field ($\Delta_{Td}$) and tetragonal distortion ($\delta_{Tetra}$) leave an A-manifold with 5 levels (see Supplementary Figure 1) with hybridized $L^z$ and $S^z$ states. When $\Delta_{Td}, \delta_{Tetra} \gg \lambda$, it leads to a pure spin $S = 2$ quintet (see Supplementary Figure 1a). Introducing a single-ion term $D(S^z)^2$ with easy-plane anisotropy, lifts the degeneracy of the quintet into levels with $S^z$ =0 (singlet), $S^z = \pm 1$ (doublet), and $S^z = \pm 2$ (doublet) where the energy-splitting between the states is given $D$ and $3D$. Supplementary Figure 1b shows the inelastic neutron scattering measured at $T$=90 K, $\sim 7 * \Theta_{CW}$ (powder averaged Curie-Weiss temperature $\Theta_{CW}\sim$-12.8 K) where the single-ion physics dominates[1]. In the spectra, two flat excitations are visible at d$E$ ~1.32 meV and 3.9 meV which indicate transitions between levels as follows $|S^z = 0\rangle \rightarrow |S^z = \pm 1\rangle$ and $|S^z = \pm 1\rangle \rightarrow |S^z = \pm 2\rangle$. Note that the transitions between $|S^z = 0\rangle$ and $|S^z = \pm 2\rangle$ are forbidden by dipole selection rules. At finite temperature, the thermal population of the spin states determines the effective spin of the system. In the low temperature region where $T \ll 3 * D$ (~45 K), the $|S^z = \pm 2\rangle$ states are depopulated and the system can then be treated as an effective $S = 1$.

## Supplementary Note 2. Analysis of a possible spin gap

To check for evidence of a gap in the spin wave dispersion at the ZC, the low energy inelastic neutron scattering was investigated using CTAX at HFIR with $E_f$=3 meV. The energy resolution of the instrument (FWHM$\sim$0.101 meV for elastic scattering) poses a challenge to directly extract a small gap due to the large scattering near the magnetic Bragg reflection at $\mathbf{Q_m}$. As an alternative approach, we examine the spin wave dispersions near the ZC and compare the calculated values with and without a small gap. The measured dispersion was obtained by fitting with a resolution convoluted Gaussian function to constant momentum transfer scans for 0.85$\leq H \leq$1.15. The extracted magnon dispersion is displayed as the contour plot along with the calculated spin waves with $\Delta_{gap} = 0$ and 0.25 meV in the Supplementary Figure 2a. The gapless Goldstone mode ($\Delta_{gap}$=0) has linear dispersion emanating from the ZC, whereas the gaped tranverse mode has a quadratic dispersion near the ZC providing a possible means of distinguising a gaped spectrum from an gapless one. To find the best description of the dispersion near the ZC, the deviation between the data and calculated dispersion is defined as $(E_{exp.} - E_{calc.})^2$. The sum of this deviation is presented in Supplementary Figure 2b as a function of the gap energy. The deviation has a minimum at $\Delta_{gap} \sim$0.25 meV, suggesting a gap in spin wave spectrum of $Ba_2FeSi_2O_7$.

The spin gap can also be extracted by extrapolating the magnetic field dependence of the $T_1$ transverse mode. The field-dependent low energy inelastic neutron scattering was measured

using the Multi-Axis Crystal Spectrometer (MACS) at NCNR with $E_f$=3 meV. The constant **Q**-linecuts were obtained at ZC with applied fields, $H$//[1 0 0], at 0, 1, 2, 3, 4, and 5 T. Supplementary Figure 2c shows the field-evolution of the spin excitation spectrum at the ZC. Each constant **Q**-linecuts were fitted to a Gaussian function to parameterize the $T_1$ transverse modes. The $T_1$ transverse mode becomes gapped by the Zeeman energy in a magnetic field, and the gap increases with the magnetic field. For a gapless system the linear extrapolation of the $T_1$-modes approaches zero energy as the field goes to zero, whereas the dispersion relation of the gapped magnon in the transverse field is described by $\omega_{mag} \propto \sqrt{\Delta_{gap}^2 + (c)^2 H^2}$, where $\Delta_{gap}$ and $c$ can be fit to the experimental data[4]. The measured $T_1$-mode dispersions were fitted to the gapless linear and gaped parabola functions, which give $\chi^2$values with 0.15 and 0.02, respectively. The lower $\chi^2$ result of the parabola function indicates the presence of a spin gap. The resulting value of the spin gap, $\Delta_{gap}$=0.18 (2) meV, is close to the value obtained from fitting to the low energy dispersion, implying that both analyses consistently indicate the presence of a small gap in the spin excitation spectrum of $Ba_2FeSi_2O_7$.

## Supplementary Note 3. Magnetic susceptibility with angular field-dependence

The angular dependent magnetic susceptibility provides evidence for the single-ion anisotropy necessary to produce a gap in the spectrum. We measured this angular dependence on a $Ba_2FeSi_2O_7$ crystal that was aligned using X-ray Laue diffraction. Magnetization was measured using vibrating sample magnetometry (VSM) implemented in a Quantum Design physical properties measurement system (PPMS-Dynacool).

Supplementary Figure 3a shows the magnetic susceptibilities, $\chi(T)$, measured with three diffrent magnetic field directions in the *ab*-plane, parallel to [1, 0, 0], [1, 1, 0], and [0, 1, 0] of crystal axes. The three $\chi(T)$ show isotropic behavior above the Néel temperature ($T_N$=5.2 K), collapsing in a single line. Noticeably, those become anisotropic with magnetic order below the $T_N$, having a weak easy-axis anisotropy along [1, 1, 0]-direction. As described in the main text, the tetragonal symmetry with $S$=2 spin allows for a single-ion anisotropy term $\mathcal{H}_A = A\sum_i [(S_i^x)^4 + (S_i^y)^4]$ ($A$>0 for easy-axis along [1, 1, 0] and [1, -1, 0]), which can induce easy-axis anisotropy in the *ab*-plane. This anisotropy term can generate a gap in the spin wave spectrum as described in Note 2.

## Supplementary Note 4. Spin wave dispersion along $L$-direction

Supplementary Figure 4 shows INS data along the $L$-direction for $[H, 0, L]$ with $H$=0 and 1, measured using HYSPEC spectrometer. The spin excitations are weakly dispersing along $L$ indicating quasi-two-dimensional behavior due to the relatively weak inter-layer coupling. As shown, the acoustic magnon ($T_1$) has a bandwidth of 0.5 meV, and the $T_2$ and $L$ modes are almost completely flat along the $L$ direction. The $L$-dependence of the spin excitations is reproduced by the GLSWT and GLSWT + one loop correction calculations with $\widetilde{J'}$=$\tilde{J}/10$.

## Supplementary Note 5. Generalized spin wave approach

In the local reference frame, the spin and quadrupolar operators can be expanded in $1/M$ as:

$$
\begin{aligned}
s_{\mathbf{r}}^{\mu} &= M\tilde{\mathcal{S}}_{00}^{\mu} + \sqrt{M} \sum_{\alpha=\pm 1} \left(\tilde{\mathcal{S}}_{\alpha 0}^{\mu} b_{\mathbf{r},\alpha}^{\dagger} + h.c.\right) \\
&+ \sum_{\alpha,\beta=\pm 1} \left(\tilde{\mathcal{S}}_{\alpha\beta}^{\mu} - \tilde{\mathcal{S}}_{00}^{\mu}\delta_{\alpha\beta}\right) \tilde{b}_{\mathbf{r},\alpha}^{\dagger}\tilde{b}_{\mathbf{r},\beta} \\
&- \frac{1}{2\sqrt{M}} \sum_{\alpha=\pm 1}\sum_{\beta=\pm 1} (\tilde{\mathcal{S}}_{\alpha 0}^{\mu}\tilde{b}_{\mathbf{r},\alpha}^{\dagger}\tilde{b}_{\mathbf{r},\beta}^{\dagger}\tilde{b}_{\mathbf{r},\beta} + h.c.) + \mathcal{O}(\frac{1}{M^{3/2}}),
\end{aligned}
\tag{S1}
$$

$$
\begin{aligned}
(s_{\mathbf{r}}^{z})^2 &= 1 - M\tilde{\mathcal{A}}_{00} - \sqrt{M} \sum_{\alpha=\pm 1} (\tilde{\mathcal{A}}_{\alpha 0}\tilde{b}_{\mathbf{r},\alpha}^{\dagger} + h.c.) \\
&- \sum_{\alpha,\beta=\pm 1} (\tilde{\mathcal{A}}_{\alpha\beta} - \tilde{\mathcal{A}}_{00}\delta_{\alpha\beta})\tilde{b}_{\mathbf{r},\alpha}^{\dagger}\tilde{b}_{\mathbf{r},\beta} \\
&+ \frac{1}{2\sqrt{M}} \sum_{\alpha=\pm 1}\sum_{\beta=\pm 1} (\tilde{\mathcal{A}}_{\alpha 0}\tilde{b}_{\mathbf{r},\alpha}^{\dagger}\tilde{b}_{\mathbf{r},\beta}^{\dagger}\tilde{b}_{\mathbf{r},\beta} + h.c.) + \mathcal{O}(\frac{1}{M^{3/2}}).
\end{aligned}
\tag{S2}
$$

The expressions for the coefficients $A_{\mathbf{k},\alpha}$ and $B_{\mathbf{k},\alpha}$ of the quadratic Hamiltonian $\mathcal{H}^{(2)}$ Eq. (17) are:

$$
\begin{aligned}
A_{\mathbf{k},+1} &= -8(x-1)x(2\tilde{J}+\tilde{J}') - (x-1)\tilde{D} \\
&\quad +2\big(x\big(1+\tilde{\Delta}\big)-1\big)\tilde{J}\gamma_{\mathbf{k}}^{xy} \\
&\quad +2\big(x\big(1+\tilde{\Delta}'\big)-1\big)\tilde{J}'\gamma_{\mathbf{k}}^{z}, \\
B_{\mathbf{k},+1} &= -2\big(x\big(\tilde{\Delta}-1\big)+1\big)\tilde{J}\gamma_{\mathbf{k}}^{xy} \\
&\quad -2\big(x\big(\tilde{\Delta}'-1\big)+1\big)\tilde{J}'\gamma_{\mathbf{k}}^{z}, \\
A_{\mathbf{k},-1} &= -16(x-1)x(2\tilde{J}+\tilde{J}') - (2x-1)D \\
&\quad -2(1-2x)^2(\tilde{J}\gamma_{\mathbf{k}}^{xy}+\tilde{J}'\gamma_{\mathbf{k}}^{z}), \\
B_{\mathbf{k},-1} &= 2(1-2x)^2(\tilde{J}\gamma_{\mathbf{k}}^{xy}+\tilde{J}'\gamma_{\mathbf{k}}^{z})
\end{aligned}
\tag{S3}
$$

**Supplementary Note 6. Cubic and cubic-linear vertices**

In this section, we derive the cubic and cubic-linear vertices given in Eq. (24) and Eq. (25). The cubic Hamiltonian has three contributions

$$
\mathcal{H}^{(3)} = \mathcal{H}_{intra}^{(3)} + \mathcal{H}_{inter}^{(3)} + \mathcal{H}_{D}^{(3)},
\tag{S4}
$$

with

$$
\begin{aligned}
\mathcal{H}_{intra}^{(3)} = \tilde{J} \sum_{\langle\mathbf{r},\mathbf{r}'\rangle,\nu} \sum_{\alpha,\beta=\pm 1} \{ &\sum_{\alpha'=\pm 1} a_{\nu}[2\tilde{\mathcal{S}}_{\alpha\beta}^{\nu}\tilde{\mathcal{S}}_{0\alpha'}^{\nu}\tilde{b}_{\mathbf{r}\alpha}^{\dagger}\tilde{b}_{\mathbf{r}\beta}\tilde{b}_{\mathbf{r}'\alpha'}] \\
&-a_{\nu}[\tilde{\mathcal{S}}_{0\alpha}^{\nu}\tilde{\mathcal{S}}_{00}^{\nu}(\tilde{b}_{\mathbf{r}\beta}^{\dagger}\tilde{b}_{\mathbf{r}\beta}\tilde{b}_{\mathbf{r}\alpha} + 2\tilde{b}_{\mathbf{r}'\beta}^{\dagger}\tilde{b}_{\mathbf{r}'\beta}\tilde{b}_{\mathbf{r}\alpha})] + h.c.\},
\end{aligned}
\tag{S5}
$$

$$
\begin{aligned}
\mathcal{H}_{inter}^{(3)} = \tilde{J}' \sum_{\langle\mathbf{r},\mathbf{r}'\rangle,\nu} \sum_{\alpha,\beta=\pm 1} \{ &\sum_{\alpha'=\pm 1} b_{\nu}[2\tilde{\mathcal{S}}_{\alpha\beta}^{\nu}\tilde{\mathcal{S}}_{0\alpha'}^{\nu}\tilde{b}_{\mathbf{r}\alpha}^{\dagger}\tilde{b}_{\mathbf{r}\beta}\tilde{b}_{\mathbf{r}'\alpha'}] \\
&-b_{\nu}[\tilde{\mathcal{S}}_{0\alpha}^{\nu}\tilde{\mathcal{S}}_{00}^{\nu}(\tilde{b}_{\mathbf{r}\beta}^{\dagger}\tilde{b}_{\mathbf{r}\beta}\tilde{b}_{\mathbf{r}\alpha} + 2\tilde{b}_{\mathbf{r}'\beta}^{\dagger}\tilde{b}_{\mathbf{r}'\beta}\tilde{b}_{\mathbf{r}\alpha})] + h.c.\},
\end{aligned}
\tag{S6}
$$

$$\mathcal{H}_D^{(3)} = \frac{\tilde{D}}{2}\sum_{\mathbf{r}}\sum_{\alpha,\beta=\pm1}[\tilde{\mathcal{A}}_{0\alpha}\tilde{b}^\dagger_{\mathbf{r}\beta}\tilde{b}_{\mathbf{r}\beta}\tilde{b}_{\mathbf{r}\alpha} + h.c.], \tag{S7}$$

To simplify the notation, we will write a particular term of (39) (in momentum space) as (I) $= \tilde{b}^\dagger_{\mathbf{q}_1,\alpha}\tilde{b}_{\mathbf{q}_2,\beta}\tilde{b}_{\mathbf{q}_3,\gamma}f(\mathbf{q}_i,t)$, with

$$f(\mathbf{q}_{1,2,3},t) = \begin{cases} 1 & t=0 \\ \gamma^{xy}_{\mathbf{q}_3} & t=1 \\ \gamma^{z}_{\mathbf{q}_3} & t=2 \end{cases}. \tag{S8}$$

The Nambu spinor of the bosonic operators can be Bogoliubov transformed into the quasi-particle representation $\vec{b}_{\mathbf{k}} = \mathcal{U}(\mathbf{k})\vec{\beta}_{\mathbf{k}}$ , where the matrix elements of $\mathcal{U}(\mathbf{k})$ are obtained from the Bogoliubov coefficients given in Eq. (20)

$$\begin{aligned}\mathcal{U}(\mathbf{k}) &= \begin{pmatrix} \mathcal{U}^{11}_{2\times2}(\mathbf{k}) & \mathcal{U}^{12}_{2\times2}(\mathbf{k}) \\ \mathcal{U}^{21}_{2\times2}(\mathbf{k}) & \mathcal{U}^{22}_{2\times2}(\mathbf{k}) \end{pmatrix} \\ &= \begin{pmatrix} u_{\boldsymbol{k},+1} & 0 & v_{\boldsymbol{k},+1} & 0 \\ 0 & u_{\boldsymbol{k},-1} & 0 & v_{\boldsymbol{k},-1} \\ v_{\boldsymbol{k},+1} & 0 & u_{\boldsymbol{k},+1} & 0 \\ 0 & v_{\boldsymbol{k},-1} & 0 & u_{\boldsymbol{k},-1} \end{pmatrix}.\end{aligned} \tag{S9}$$

After applying the above-mentioned Bogoliubov transformation, we obtain

$$\begin{aligned}(\mathrm{I}) = \sum_{n_{1,2,3}} \{ & F^{(a)}(\alpha\beta\gamma, n_{1,2,3}; \mathbf{q}_{1,2,3}, t)\beta_{\mathbf{q}_1,n_1}\beta_{\mathbf{q}_2,n_2}\beta_{\mathbf{q}_3,n_3} \\ & + F^{(b)}(\alpha\beta\gamma, n_{1,2,3}; \mathbf{q}_{1,2,3}, t)\beta^\dagger_{\bar{\mathbf{q}}_1,n_1}\beta^\dagger_{\bar{\mathbf{q}}_2,n_2}\beta^\dagger_{\bar{\mathbf{q}}_3,n_3} \\ & + F^{(c)}(\alpha\beta\gamma, n_{1,2,3}; \mathbf{q}_{1,2,3}, t)\beta^\dagger_{\bar{\mathbf{q}}_1,n_1}\beta^\dagger_{\bar{\mathbf{q}}_2,n_2}\beta_{\mathbf{q}_3,n_3} \\ & + F^{(d)}(\alpha\beta\gamma, n_{1,2,3}; \mathbf{q}_{1,2,3}, t)\beta^\dagger_{\bar{\mathbf{q}}_1,n_1}\beta_{\mathbf{q}_2,n_2}\beta_{\mathbf{q}_3,n_3} \\ & + L^{(c)}(\alpha\beta\gamma, n_{1,2,3}; \mathbf{q}_{1,2,3}, t)\delta_{\mathbf{q}_3,\bar{\mathbf{q}}_2}\delta_{n_3,n_2}\beta^\dagger_{\bar{\mathbf{q}}_1,n_1} \\ & + L^{(d)}(\alpha\beta\gamma, n_{1,2,3}; \mathbf{q}_{1,2,3}, t)\delta_{\mathbf{q}_2,\bar{\mathbf{q}}_1}\delta_{n_2,n_1}\beta_{\mathbf{q}_3,n_3} \}.\end{aligned} \tag{S10}$$

The explicit forms of $F^{(a,b,c,d)}$ and $L^{(c,d)}$ can be obtained by simple algebras, which are not shown here for the sake of brevity.

The "sink" ("source") function $F^{(a)}$ ($F^{(b)}$) is symmetric under permutations of all three legs (momenta and flavors). Consequently, we introduce the symmetrized functions

$$\tilde{F}^{(a)} \equiv \sum_{P(\mathbf{q}_{1,2,3};n_{1,2,3})} F^{(a)}, \qquad \tilde{F}^{(b)} \equiv \sum_{P(\mathbf{q}_{1,2,3};n_{1,2,3})} F^{(b)}. \tag{S11}$$

Similarly, the "decay" function $F^{(c)}$ and the "fusion" function $F^{(d)}$ are symmetrized for the two outgoing and the two incoming legs, respectively,

$$\tilde{F}^{(c)} = \sum_{P(\mathbf{q}_{1,2};n_{1,2})} F^{(c)}, \qquad \tilde{F}^{(d)} = \sum_{P(\mathbf{q}_{2,3};n_{2,3})} F^{(c)}. \tag{S12}$$

After inserting the above results into Eq. (39), we obtain the explicit forms of the cubic vertices in $V_{s/d}^{(3)}$ Eq. (24) and $V_{\alpha}^{L}$ in Eq. (25).

**Supplementary Note 7. Quartic vertex**

The quartic contributions to the expansion (9) are

$$\mathcal{H}^{(4)} = \mathcal{H}_{intra}^{(4)} + \mathcal{H}_{inter}^{(4)}, \tag{S13}$$

with

$$\begin{aligned}\mathcal{H}_{\text{intra}}^{(4)} = \tilde{J} \sum_{\langle \mathbf{r},\mathbf{r}' \rangle,\nu} \sum_{\alpha,\beta=\pm 1} &\{[a_\nu \tilde{S}_{00}^{\nu} \tilde{S}_{00}^{\nu} \tilde{b}_{\mathbf{r}\alpha}^{\dagger} \tilde{b}_{\mathbf{r}'\beta}^{\dagger} \tilde{b}_{\mathbf{r}\alpha} \tilde{b}_{\mathbf{r}'\beta}] \\ &+ \sum_{\alpha'\beta'=\pm 1} \left[a_\nu \tilde{S}_{\alpha\beta}^{\nu} \tilde{S}_{\alpha'\beta'}^{\nu} \tilde{b}_{\mathbf{r}\alpha}^{\dagger} \tilde{b}_{\mathbf{r}'\alpha'}^{\dagger} \tilde{b}_{\mathbf{r}\beta} \tilde{b}_{\mathbf{r}'\beta'}\right] \\ &-2 \sum_{\alpha'=\pm 1} \left[a_\nu \tilde{S}_{\alpha\beta}^{\nu} \tilde{S}_{00}^{\nu} \tilde{b}_{\mathbf{r}\alpha}^{\dagger} \tilde{b}_{\mathbf{r}'\alpha'}^{\dagger} \tilde{b}_{\mathbf{r}\beta} \tilde{b}_{\mathbf{r}'\alpha'}\right] \\ &- \sum_{\alpha'=\pm 1} \left[a_\nu \tilde{S}_{\alpha 0}^{\nu} \tilde{S}_{\beta 0}^{\nu} \tilde{b}_{\mathbf{r}\alpha}^{\dagger} \tilde{b}_{\mathbf{r}'\beta}^{\dagger} \tilde{b}_{\mathbf{r}'\alpha'}^{\dagger} \tilde{b}_{\mathbf{r}'\alpha'} + h.c.\right] \\ &- \sum_{\alpha'=\pm 1} [a_\nu \tilde{S}_{\alpha 0}^{\nu} \tilde{S}_{0\beta}^{\nu} \tilde{b}_{\mathbf{r}\alpha}^{\dagger} \tilde{b}_{\mathbf{r}'\alpha'}^{\dagger} \tilde{b}_{\mathbf{r}'\alpha'} \tilde{b}_{\mathbf{r}'\beta} + h.c.]\}.\end{aligned} \tag{S14}$$

Similarly to the cubic contribution, $\mathcal{H}_{inter}^{(4)}$ can be obtained from $\mathcal{H}_{intra}^{(4)}$ by substituting $\tilde{J} \to \tilde{J}'$, $a_\nu \to b_\nu$. The matrix elements appear in the normal ordering of the quartic vertex are defined as:

$$\begin{aligned}\bar{N}_{\mathbf{r}\mathbf{r}'}^{\alpha\beta} &\equiv \frac{1}{N} \sum_{\langle \mathbf{r},\mathbf{r}' \rangle} \langle b_{\mathbf{r}\alpha}^{\dagger} b_{\mathbf{r}'\beta} \rangle \\ &= \frac{1}{N} \sum_{\mathbf{k}} \sum_{n} \mathcal{U}_{\alpha,n}^{21}(\mathbf{k}) [\mathcal{U}_{\beta,n}^{21}(\mathbf{k})]^{*} \cos\left[\mathbf{k} \cdot (\mathbf{r}' - \mathbf{r})\right], \\ \Delta_{\mathbf{r}\mathbf{r}'}^{\alpha\beta} &\equiv \frac{1}{N} \sum_{\langle \mathbf{r},\mathbf{r}' \rangle} \langle b_{\mathbf{r}\alpha} b_{\mathbf{r}'\beta} \rangle \\ &= \frac{1}{N} \sum_{\mathbf{k}} \sum_{n} \mathcal{U}_{\alpha,n}^{11}(\mathbf{k}) [\mathcal{U}_{\beta,n}^{21}(\mathbf{k})]^{*} \cos\left[\mathbf{k} \cdot (\mathbf{r}' - \mathbf{r})\right], \\ \bar{\Delta}_{\mathbf{r}\mathbf{r}'}^{\alpha\beta} &\equiv \frac{1}{N} \sum_{\langle \mathbf{r},\mathbf{r}' \rangle} \left\langle b_{\mathbf{r}\alpha}^{\dagger} b_{\mathbf{r}'\beta}^{\dagger} \right\rangle \\ &= \frac{1}{N} \sum_{\mathbf{k}} \sum_{n} \mathcal{U}_{\alpha,n}^{21}(\mathbf{k}) [\mathcal{U}_{\beta,n}^{11}(\mathbf{k})]^{*} \cos[\mathbf{k} \cdot (\mathbf{r}' - \mathbf{r})],\end{aligned} \tag{S15}$$

We note that some of these matrix elements are equal to zero because of the residual U(1) symmetry of the antiferromagnetic order. To obtain the normal-ordered Hamiltonian Eq. (34), we apply a mean-field (Hartree-Fock) decoupling to the quartic Hamiltonian Eq. (42), for example,

$$\tilde{b}_{\mathbf{r}\alpha}^{\dagger} \tilde{b}_{\mathbf{r}'\beta}^{\dagger} \tilde{b}_{\mathbf{r}\alpha} \tilde{b}_{\mathbf{r}'\beta} \simeq \Delta_{\mathbf{r}\mathbf{r}'}^{\alpha\beta} \tilde{b}_{\mathbf{r}\alpha}^{\dagger} \tilde{b}_{\mathbf{r}'\beta}^{\dagger} + \bar{N}_{\mathbf{r}'\mathbf{r}'}^{\beta\beta} \tilde{b}_{\mathbf{r}\alpha}^{\dagger} \tilde{b}_{\mathbf{r}\alpha} \tag{S16}$$

$$+\bar{N}^{\beta\alpha}_{\mathbf{r'r}}\tilde{b}^{\dagger}_{\mathbf{r}\alpha}\tilde{b}_{\mathbf{r}\beta}+\bar{\Delta}^{\alpha\beta}_{\mathbf{rr'}}\tilde{b}_{\mathbf{r}\alpha}\tilde{b}_{\mathbf{r'}\beta}$$
$$+\bar{N}^{\alpha\alpha}_{\mathbf{rr}}\tilde{b}^{\dagger}_{\mathbf{r'}\beta}b_{\mathbf{r'}\beta}+\bar{N}^{\alpha\beta}_{\mathbf{rr'}}\tilde{b}^{\dagger}_{\mathbf{r'}\beta}\tilde{b}_{\mathbf{r}\alpha}.$$

The coefficients $V^{(4,N)}_{\alpha\alpha}$ that appear in the normal term of Eq. (34) can be derived after consecutive Fourier and Bogoliubov transformations.

## Supplementary Note 8. One-loop diagrams in the long-wavelength limit

Without loss of generality, we consider an isotropic Heisenberg model, i.e. $\tilde{J}=\tilde{J}'$, $\tilde{\Delta}=\tilde{\Delta}'$ to show the $1/\mathbf{q}$ divergence of the one-loop diagrams involving the Goldstone mode. According to Eq. (20),

$$\lim_{\mathbf{q}\to 0} u_{\mathbf{q},+}, v_{\mathbf{q},+} = \sqrt{\frac{\tilde{J}d}{v_{\mathbf{0},+}}\frac{1}{\sqrt{q}}}, -\sqrt{\frac{\tilde{J}d}{v_{\mathbf{0},+}}\frac{1}{\sqrt{q}}} \quad \text{(S17)}$$

where $v_{\mathbf{0},+}=2\tilde{J}d\sqrt{\tilde{D}/(4\tilde{J}d^2)+1/d}$ is the spin wave velocity of the Goldstone mode and $d=3$ is the spatial dimension of the lattice equal to half of the coordination number. Note that the cubic vertices are proportional to a product of the Bogoliubov coefficients of three legs

$$V^{(3)}_{d,s}\propto u(v)_{\mathbf{q}_1,\alpha}u(v)_{\mathbf{q}_2,\beta}u(v)_{\mathbf{q}_3,\gamma}. \quad \text{(S18)}$$

For the decay and sink diagrams shown on the second line of figure 5a in the main text, we can choose, for instance, $\mathbf{q}_3=\mathbf{q}\sim\mathbf{0}$, $\gamma=+1$ to contract with the leg of the long-wavelength bosons. Consequently,

$$\Sigma^{(d,s)}(+)\sim|V^{(3)}_{d,s}(\mathbf{q}_{1,2},\mathbf{q};\alpha\beta+)|^2\sim(1/\sqrt{\mathbf{q}})^2\sim 1/q. \quad \text{(S19)}$$

As for the cubic-linear diagrams, we need to choose two legs to contract with the long-wavelength boson, implying that

$$\Sigma^{(cl)}(+)\sim V^{(3)}_{d}(\mathbf{0},-\mathbf{q}\mathbf{q};-++)\sim 1/q. \quad \text{(S20)}$$

Finally, notice that $V^{(4,N)}_{++}\sim 1/q$ in the long-wavelength limit, because the quadratic forms of the transverse boson in Eq. (43) after the Bogoliubov transformation are proportional to $u(v)_{\mathbf{q},+1}u(v)_{\mathbf{q},+1}$. By adding up all diagrams in $\mathcal{O}(M^0)$, we have verified that the coefficient of the $1/q$-factor vanishes, implying that the Goldstone mode is preserved after the one-loop correction.

## Supplementary Note 9. Calculation of the inelastic neutron scattering intensity

The imaginary-time dynamical spin susceptibility is defined as:

$$\chi^{\mu\nu}(\mathbf{q},i\omega_n)=-\int_0^\beta d\tau e^{i\omega_n\tau}\langle\mathcal{T}_\tau\left[s^\mu_{\mathbf{q}}(\tau)s^\nu_{\bar{\mathbf{q}}}(0)\right]\rangle. \quad \text{(S21)}$$

The *real-time* spin-spin correlation function in Eq. (22) is obtained by using the fluctuation-dissipation theorem at $T=0$ after the analytic continuation $i\omega_n\to\omega+i0^+$:

$$S^{\mu\nu}(\mathbf{q},\omega)=-2\Im[\chi^{\mu\nu}(\mathbf{q},\omega)]. \quad \text{(S22)}$$

Up to order $\mathcal{O}(1/M)$, $S^{\mu\nu}$ acquires two contributions:

$$S^{\mu\nu} = S^{\mu\nu}_{\mathrm{qp}} + S^{\mu\nu}_{\mathrm{tc}}, \tag{S23}$$

where $S^{\mu\nu}_{\mathrm{qp}}$ includes contributions from the quasi-particle channel associated with "transverse fluctuations" of the SU(3) order parameter, while $S^{\mu\nu}_{\mathrm{tc}}$ includes two-particle contributions associated with "longitudinal fluctuations" of the SU(3) order parameter[3]. The latter is not analyzed in this work, as it only contributes to the continuum. After the one-loop corrections, the quasi-particle channel can be written as a linear combination of the dressed bosonic propagators $\mathcal{G}$. For details, see Ref. 3.

To explain the experimental data, we used the Lorentzian broadening line-shape on the calculation, which is naturally implemented from the Green's functions incorporated by simply adding an imaginary part to the real frequency: $\omega \rightarrow \omega + i\eta$. Since the instrumental resolution of the INS spectrum is conventionally modeled with a Gaussian function, here we confirm the validity of using a Lorentzian broadening in our analysis. Supplementary Figure 5a compares the resolution convoluted Gaussian and Lorentzian broadening for the same calculated energy scan at the ZC ($\mathbf{Q} = \mathbf{Q_m}$). For clearer comparison, the $T_1$ transverse mode was subtracted in the calculations. The comparison shows a discrepancy in the tail of the $T_2$-mode, however, the $L$-mode has nearly identical linewidths and peak-areas for both broadenings, which confirms that the Lorentzian and Gaussian broadenings essentially give the same result. Also, this result shows that the slightly extended tail of $T_2$-mode does not affect the line shape of the $L$-mode at the ZC. Therefore, we conclude that the Lorentzian broadening provides a good approximation of the resolution function in the present case.

## Supplementary Note 10. Longitudinal mode extraction at the ZC

Since the $L$-mode is close to the large quasi-elastic spectral weight in the proximity of the magnetic Bragg peak at the ZC ($\mathbf{Q} = \mathbf{Q_m}$), the modeling of this scattering may affect the extraction of the accurate peak position of the $L$-mode. We note that a gaped $T_1$-transverse mode can further complicate the determination of the quasi-elastic line-shape. A single Gaussian (Lorentzian) function underestimate (overestimate) the line width. Alternatively, we modeled the peak with the Gaussian + Lorentzian functions. Here the Gaussian peak describes the elastic scattering and the Lorentzian peak with its detailed balance describe the inelastic contribution. Supplementary Figure 5b shows the fitting the function to the data (cyan solid line). The longitudinal- (transverse-) mode was extracted from the remaining spectral weight, which gives the peak center at 1.30 meV with FWHM=0.644 meV (2.53 meV with FWHM=0.23).

## Supplementary Note 11. Fitting the INS spectrum.

Since the one-loop corrections involve the evaluation of several numerical integrations (cf. Eqs. (25, 32, 33) and Eq. (S15)), and the many-body effects are stronger near the zone center, we adopted the criterion of reducing the number of free model parameters to a minimum value. In addition, the splitting of the transverse modes and the longitudinal mode is most striking near the ZC and the calculated spectrum has a strong dependence on $\tilde{J}$ and $\tilde{D}$, while it is much less sensitive to $\tilde{\Delta}$ and $\tilde{\Delta}'$. Moreover, as shown in the Supplementary Figure 4a and 4d, the spin excitations along the $L$-direction are almost flat, indicating that the inter-layer exchange constant is relatively small. We then choose $\tilde{J}$ and $\tilde{D}$ as free parameters while fixing $\tilde{\Delta} = \tilde{\Delta}' = 1/3$ (isotropic exchange

interaction $\Delta = \Delta' = 1$ in the $S = 2$ model Eq. (1)) and $\tilde{J}' = 0.1\tilde{J}$. The parameter set $\mathcal{A}$ (GLSWT) is obtained by fitting the energy of the longitudinal- and transiverse modes at the ZC with the analytical expression given in Eq. (21) (see Note 10 for the experimental energy values). The fitting procedue becomes more challenging upon inclusion of the one loop corrections because the calculation involves multiple integrations and the renormalization in the Hamiltonian parameters turns out to be rather strong for $Ba_2FeSi_2O_7$. Here, we simply compute the renormalization of the real part of the self-energy for both modes at the ZC and then deform the set of parameters $\mathcal{A}$ (GLSWT) until the renormalized peaks positions match with the experimental peak positions at the ZC. The best-fit parameter set is listed as set $\mathcal{B}$ in Table 1. The errors of these parameters were estimated from the uncertainty in the $\chi^2$ value[4] defined by $\chi^2 = \frac{(E_L^{exp.} - E_L^{cal.})^2}{\sigma_{std.dev.}^2} + \frac{(E_{T2}^{exp.} - E_{T2}^{cal.})^2}{\sigma_{std.dev.}^2}$, where $E_i^{exp.}$, $E_i^{cal.}$, $\sigma_{std.dev.}$($i$=$L$, $T_2$) corrrespond to the energy and the standard deviation of the modes at the ZC.

**Supplementary Note 12. Ordered moment**

To have an independent validation of the model parameters obtained from fits of the INS data, we also compare the calculated staggered magnetic moment $M_S$ using the_set $\mathcal{B}$ given in Table 1 with the value of 2.95 $\mu_B$ that was extracted from the neutron diffraction experiment[1]. At the mean-field level, the effective $S = 1$ model predicts $M_S = g_{ab}\ \sqrt{3}\ |\langle s_{\mathbf{r}}^x \rangle|\ \mu_B =\ 2.92\ \mu_B$ for $g_{ab}\ = 2.18$ [1], while the $1/M$ correction from the GLSWT yields $M_S = 2.79\ \mu_B$, where the factor $\sqrt{3}$ arises from $P_{S=1} S_{\mathbf{r}}^x P_{S=1} = \sqrt{3} s_{\mathbf{r}}^x$. We note that the relatively smaller calculated value of $M_S$ can be attributed to the fact that it is calculated from the effective $S = 1$ model. To verify this argument, we performed the mean field calculation using the original $S = 2$ model Eq. (1) and obtained $M_S = 3.09\ \mu_B$. We further infer that the $1/M$ correction, which corresponds to an SU(5) GLSWT calculation for $S = 2$, will bring the calculated value very close to the measured one (for a relative moment reduction equal to the one obtained for the SU(3) GLSWT the result is $M_S = 2.95\ \mu_B$ in good agreement with the measured value). This agreement confirms the validity of the spin one model obtained from fits of the INS data with the GLSWT plus one-loop corrections. The small reduction (-4.5%) of $M_S$, relative to the mean field value, indicates that the assumption of validity of a perturbative $1/M$-expansion is self-consistently verified for the Hamiltonian parameters of $Ba_2FeSi_2O_7$.

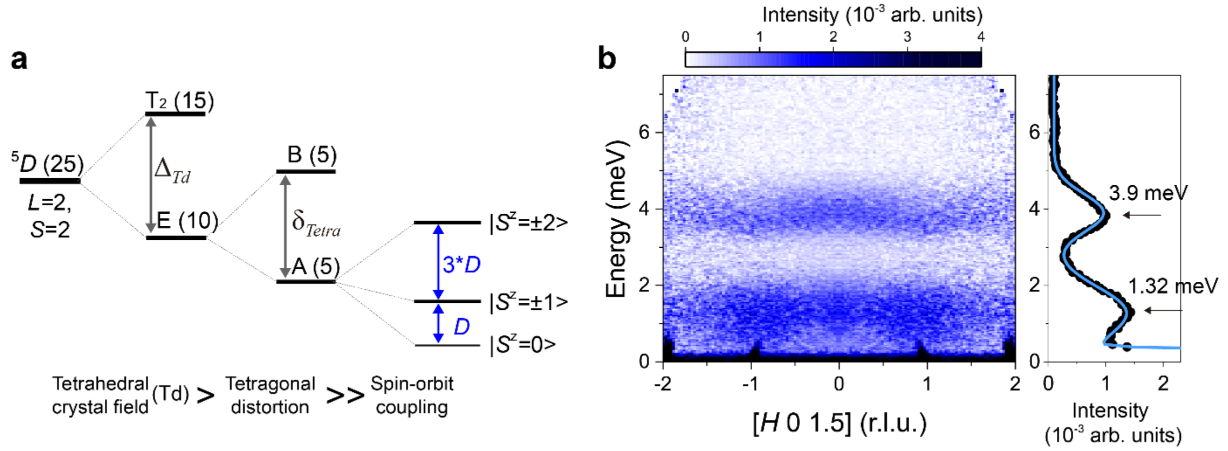


**Supplementary Figure 1. Orbital configuration and single-ion excitation of $Ba_2FeSi_2O_7$**

**a** Orbital energy states of $Fe^{2+}$ with a tetrahedral crystal field ($\Delta_{Td}$), tetragonal distortion ($\delta_{Tetra}$), and spin orbit coupling ($\lambda$). **b** The left panel shows inelastic neutron scattering data measured at $T$=90 K, symmetrized over negative and positive $H$ and integrated over $L$=[0.9, 2.1] and $K$=[-0.1, 0.1]. The integrated scattering intensity over $H$=[-2, 2] is shown in the right panel. The two peaks were fitted with Gaussian functions (solid blue line). Arrows indicate the peak centers at 1.32 meV and 3.9 meV.

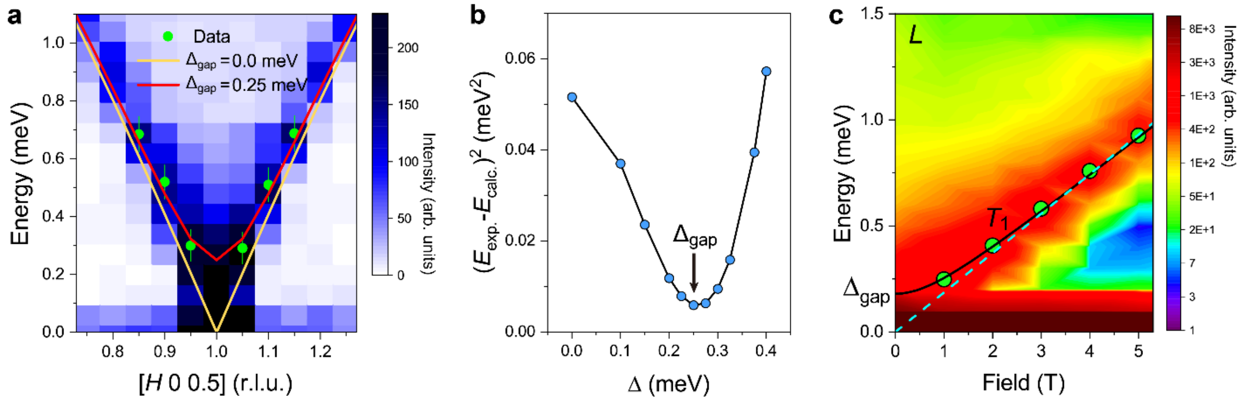


**Supplementary Figure 2. Gap excitation at magnetic zone center**

**a** Contour map for low energy inelastic neutron scattering data near the zone center. The magnon dispersion extracted from the data is indicated by green circles and is compared with the dispersion calculated with the GLSWT with and without a gap ($\Delta_{\mathrm{gap}}$ =0.25 meV) in the spectrum. **b** Sum of the square of the energy difference between the measured and calculated dispersion as a function of gap size. The arrow marks the gap size $\Delta_{\mathrm{gap}} \sim 0.25$ meV that best fits the data. **c** Contour plot for the field-dependent inelastic neutron scattering at ZC. The cyan circles indicate the energy of $T_1$ transverse modes. '$L$' denotes the scattering from the longitudinal mode. The $T_1$-modes were fitted to gapless linear function (dashed cyan line) and gapped parabola function (solid black line), $\omega_{mag} = \sqrt{\Delta_{gap}^2 + (c)^2 H^2}$. Fitting to the gapped parabola function gives a gap with 0.18 (2) meV.

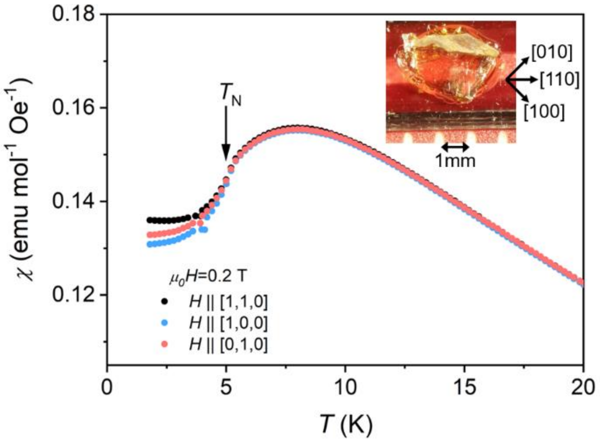


**Supplementary Figure 3. Magnetic susceptibility with angular field-dependence in *ab*-plane.** Magnetic susceptibilities measured with magnetic fields (*H*=0.2 T) along *H*||[1, 0, 0], [1, 1, 0], and [0, 1, 0] directions of the crystal structure. The inset shows a picture of the measured $Ba_2FeSi_2O_7$ single crystal with the crystal orientations.

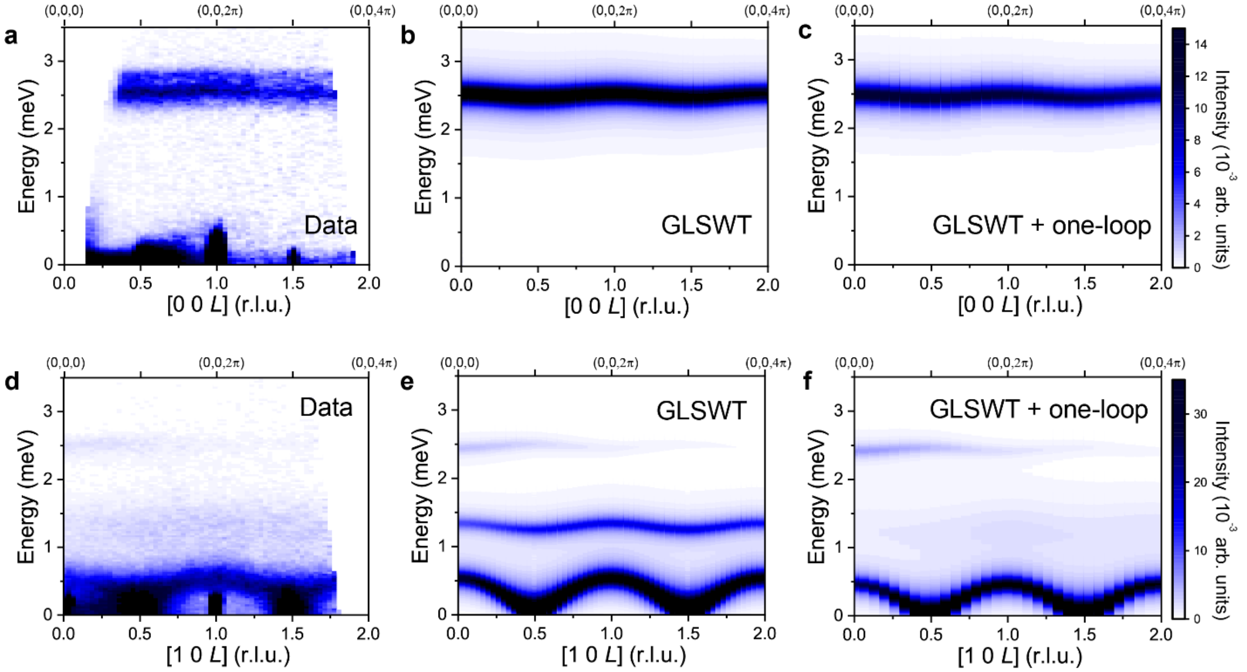


**Supplementary Figure 4. Spin excitation along *L*-direction**

Inelastic neutron scattering spectra along $[H, 0, L]$ for $H$=0 (**a-c**) and 1 (**d-f**) along with the calculated spectra using the GLSWT and GLSWT+one-loop corrections using parameter sets $\mathcal{A}$ and $\mathcal{B}$ in Table. 1, respectively. All the calculated spectra were convoluted with the instrumental resolution of HYSPEC.

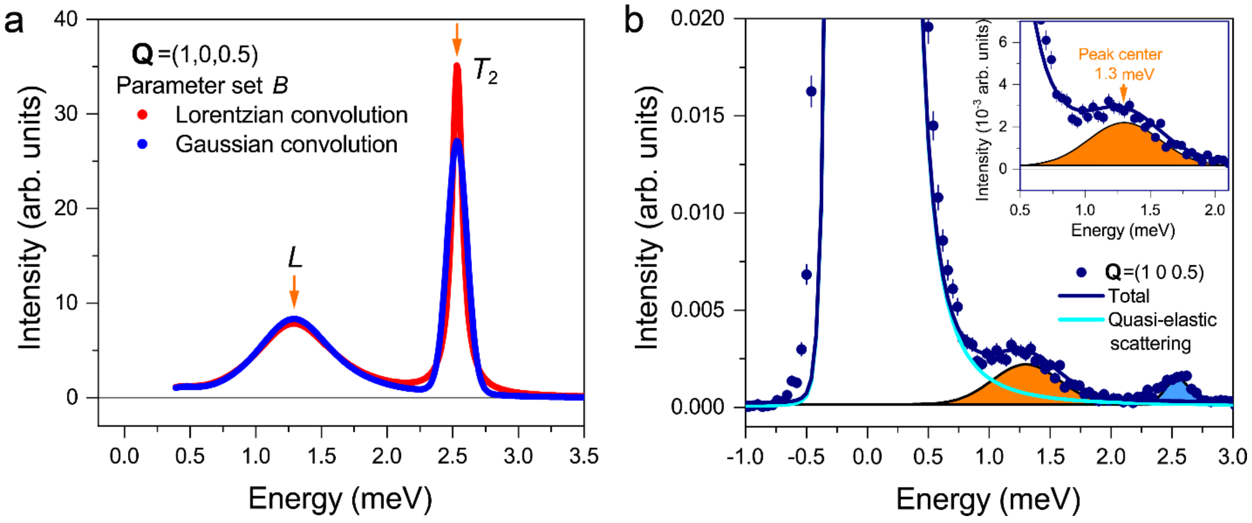


**Supplementary Figure 5. Line broadening of the calculated spectrum and the extraction of the longitudinal mode at the ZC.**
**a** Calculated INS intensity of GLSWT+one-loop correction model after applying the Lorentzian and Gaussian broadening (convolution). $L$ ($T_2$) indicates the longitudinal (transverse) mode. **b** Constant momentum cut at the ZC ($\mathbf{Q} = \mathbf{Q_m}$), measured using HYSPEC at SNS, shows a large elastic scattering, quasi-elastic scattering, $L$, and $T_2$ modes. The large elastic and quasi-elastic scattering are fitted with Gaussian + Lorentzian functions (solid cyan line). The orange- and blue-shaded regions indicate $L$- and $T_2$-modes extracted from fitting.

## References


1. Jang T-H, *et al.* Physical properties of a quasi-two-dimensional square lattice antiferromagnet $Ba_2FeSi_2O_7$. Preprint at https://arxiv.org/abs/2108.00999 (2021).
2. Mai TT, *et al.* Terahertz spin-orbital excitations in the paramagnetic state of multiferroic $Sr_2FeSi_2O_7$ *Phys Rev B* **94**, (2016).
3. Mourigal M, Fuhrman WT, Chernyshev AL, Zhitomirsky ME. Dynamical structure factor of the triangular-lattice antiferromagnet. *Phys Rev B* **88**, 094407 (2013).
4. Bevington PR, Robinson DK. *Data reduction and error analysis*. McGraw Hill, New York (2003).
